# Interactive Mobile Phone HIV Adherence Support for Men Who Have Sex With Men in the Philippines Connect for Life Study: Mixed Methods Approach to Intervention Development and Pilot Testing

**DOI:** 10.2196/30811

**Published:** 2022-02-03

**Authors:** Cara O'Connor, Katerina Leyritana, Aoife M Doyle, James J Lewis, Randeep Gill, Edsel Maurice Salvaña

**Affiliations:** 1 Faculty of Epidemiology and Population Health London School of Hygiene and Tropical Medicine London United Kingdom; 2 Sustained Health Initiatives of the Philippines (SHIP) Mandaluyong Philippines; 3 Medical Research Council International Statistics and Epidemiology Group London School of Hygiene and Tropical Medicine London United Kingdom; 4 Y Lab, The Public Services Innovation Lab for Wales School of Social Sciences Cardiff University Cardiff United Kingdom; 5 Johnson & Johnson Global Public Health London United Kingdom; 6 Institute of Molecular Biology and Biotechnology National Institutes of Health University of the Philippines Manila Ermita Philippines; 7 Division of Infectious Diseases (Global Health) Department of Medicine University of Pittsburgh Pittsburgh, PA United States

**Keywords:** mHealth, adherence, HIV, antiretroviral therapy, intervention development, mobile phone

## Abstract

**Background:**

The HIV epidemic in the Philippines is one of the fastest growing epidemics globally, and infections among men who have sex with men are rising at an alarming rate. The World Health Organization recommends the use of mobile health (mHealth) technologies to engage patients in care and ensure high levels of adherence to antiretroviral therapy (ART). Existing mHealth interventions can be adapted and tailored to the context and population served.

**Objective:**

This study aims to create a locally tailored intervention using a mobile phone platform to support treatment adherence for HIV patients on ART in the Philippines.

**Methods:**

A mixed methods approach guided by the Behavior Change Wheel framework was used to adapt an existing mHealth adherence support platform for the local setting and target population. A literature review, retrospective clinical record review, and focus group discussions with patients were conducted to understand the drivers of ART adherence and tailor the intervention accordingly. The resulting intervention was pilot-tested for 8 weeks, followed by focus group discussions with patients who received the intervention to assess the acceptability of the design.

**Results:**

Key issues contributing to nonadherence included side effects, lack of behavioral skills for pill taking, social support, mental health, and substance use. Patients identified mHealth as an acceptable mode of intervention delivery and wanted mHealth services to be highly personalizable. The study team, clinicians, and software developers integrated these findings into the intervention, which included a menu of services as follows: pill reminders, health tips, adherence feedback, appointment reminders, and symptom reporting. During the pilot phase, technical issues in the interactive voice response system (IVRS) were identified and addressed. Patients who participated in the pilot phase expressed a preference for SMS text messaging over the IVRS. Patients responded positively to the appointment reminders and health tips, whereas patient feedback on daily and weekly pill reminders and adherence feedback was mixed.

**Conclusions:**

The mobile phone–based SMS text messaging and IVRS intervention was acceptable to men who have sex with men in Manila, the Philippines, and qualitative analysis suggested that the intervention helped promote ART adherence and appointment attendance.

## Introduction

### HIV on the Rise in the Philippines

The Philippines has the fastest growing HIV epidemic in the Asia-Pacific region [1-3]. National surveillance data show that the number of new HIV cases in the Philippines has increased at an alarming rate during the past decade, with an increase from 311 cases identified in 2007 to 12,778 cases identified in 2019—a 41-fold increase in new HIV diagnoses [4]. According to the surveillance reports by the Joint United Nations Program on HIV/AIDS, the Philippines’ progress toward reaching HIV/AIDS 90-90-90 goals has been slow, with 73% of people living with HIV being aware of their status, 44% on treatment, and low coverage of viral load testing (<50%) [5,6].

The group most impacted by HIV in the Philippines is men who have sex with men (MSM), representing 84% of new diagnoses since 2015. The median age of new cases is 28 years, and >80% of people living with HIV/AIDS in the Philippines are aged <35 years [4,7]. In 2015, a national surveillance survey found that HIV prevalence among MSM who practiced anal sex was 6%—an increase from 3.3% in 2013 [4-6,8-10].

As the burden of HIV increases, it is imperative that as many HIV-infected people as possible are diagnosed, started on treatment, and successfully retained in care. Achieving adequate viral suppression through the use of antiretroviral therapy (ART) will be one of the key tools in ending the HIV epidemic in the Philippines. Unfortunately, widespread stigma, lack of knowledge, and barriers to accessing care pose a challenge to engaging patients in testing and ensuring high levels of adherence to ART and retention in care [8,10,11]. As in many low- and middle-income countries, high rates of first-line treatment failure, loss to follow-up, and suboptimal treatment adherence led to poor outcomes for many HIV patients in the Philippines [12,13].

Evidence-based public health interventions are required. However, a 2015 report by the World Health Organization highlights that the body of HIV research conducted in the Philippines has been limited [14], and a systematic review of HIV risk studies in the Philippines through April 2018 found only 3 publications that included data about the group most affected by HIV—MSM [15].

### Mobile Health for Adherence

As mobile phone technologies have become widespread in low- and middle-income countries, mobile phone interventions have become increasingly popular in the global health and development sectors as a potentially inexpensive and efficient way to communicate with and deliver services to people.

Mobile phones are multifunctional tools that can be used for a variety of functions that range from simple alarm functions, SMS text messaging, calls, interactive voice response systems (IVRSs), and complex apps and games. People usually have their mobile devices with them; therefore, using mobile technologies allows the timing of the intervention delivery to be synchronized with the most relevant time to claim the attention of the recipient [16]. Moreover, mobile phones can be used almost anywhere. As wireless coverage is improving globally, mobile communications can be provided even in remote areas.

In the Philippines, 99% of the population is reached by mobile cellular network coverage and mobile phone use is among the highest in the world, with 155 mobile connections per 100 people [17,18]. Although the coverage of mobile networks is high, smartphone coverage and mobile internet (Long-Term Evolution) speeds are lower in the Philippines than in other countries in the region [19], which limits the potential reach of mobile internet and app-based solutions.

The 2016 World Health Organization Consolidated Guidelines on the Use of ART for the Treatment and Prevention of HIV Infection promoted the use of SMS text messaging to improve adherence to therapy [20]. Research has shown that mobile health (mHealth) interventions have potential benefits for a wide variety of health issues, including antiretroviral adherence, smoking cessation, diabetes control, maternal health, and vaccination programs [16,21].

Mobile phone interventions have proven successful in improving ART adherence in Africa, South Asia, and Latin America [22-28]. Several systematic reviews have been published regarding mHealth for ART adherence specifically [29,30]. A variety of mHealth approaches to improve adherence to antiretroviral medications have been studied globally, including daily and weekly short text messages [22,31-33], weekly long text messages [31], weekly voice messages [34], and fortnightly phone calls [35,36]. The outcome measures of ART adherence interventions vary; outcome measures include self-reported adherence, objective measures of adherence (ie, pill count, pharmacy refill, and medication monitors), biological end points (ie, viral load suppression), and quality-of-life measures.

In Kenya, 2 important examples of successful ART adherence interventions were implemented. At the WelTel Kenya1 multisite randomized clinical trial of HIV-infected adults initiating ART, adherence to ART was reported in 61.5% (168/273) of patients receiving the SMS text messaging intervention compared with 49.8% (132/265) of patients in the control group (relative risk for nonadherence 0.81; *P*=.006). Suppressed viral loads were reported in 57.1% (156/273) of patients in the SMS text messaging group and 48.3% (128/265) of patients in the control group (relative risk for virologic failure 0.84; *P*=.04) [22].

Another randomized trial of 131 adult patients who had initiated ART less than 3 months before enrollment found that 53% of participants receiving weekly SMS text messaging reminders achieved adherence of ≥90% during the 48 weeks of the study, compared with 40% of participants in the control group (*P*=.03). Participants in groups receiving weekly reminders were also significantly less likely to experience treatment interruptions exceeding 48 hours during the 48-week follow-up period compared with participants in the control group [31].

Multiple reviews of the literature on adherence programs suggest that mobile phones are a feasible, acceptable, and effective mode of delivery for HIV interventions targeting young MSM [37-40]. There is also evidence that daily reminders can support habit forming over 2-3 months and that weekly reminders effectively support adherence [23,25,29,41,42]. It is not clear whether improvements in adherence are sustained if reminders are stopped once a habit is formed. Some evidence suggests that weekly messages with interactive elements that elicit a response from the user may be the most effective SMS text messaging adherence interventions, but many questions remain unanswered [23,24].

### Aim and Objective

The investigators aim to create a locally tailored intervention using a mobile phone platform to support treatment adherence for HIV patients on ART at the study clinic in Metro Manila, Philippines.

The objective of the formative research phase of the study is to adapt an existing technology platform (Connect for Life) for the local context. We seek to answer the following questions:

- What is the level of adherence in the study clinic population and similar populations in the country and region?
- What are the barriers to and determinants of ART adherence among the study clinic population?
- What components should an mHealth intervention include to address these barriers and determinants?

## Methods

### Setting

The Sustained Health Initiatives of the Philippines (SHIP) Clinic is a public-private partnership that opened in 2012. It is a low-cost, private facility in Metro Manila, a city of approximately 13 million people in the predominantly Catholic country of the Philippines and the most densely populated city in the world. As of April 2021, the SHIP clinic provided HIV primary care and wraparound services to approximately 900 patients. Between 2012 and 2018, SHIP was a satellite partner clinic of the Sexually Transmitted Infection/AIDS Guidance Intervention and Prevention Unit at the Philippine General Hospital, the largest public hospital in the country.

Approximately 98% of SHIP’s clients are MSM, with an average age of 30 years at the initial consultation. Most are employed full time or part time. The patients come from all regions of Metro Manila, and some live outside of Metro Manila in other provinces. SHIP currently enrolls approximately 4 new patients each month.

Ethical clearance for the study was obtained from the University of the Philippines Manila Research Ethics Board (protocol number 2016-265-01) and from the London School of Hygiene and Tropical Medicine (reference number 11631). All patients provided written consent before inclusion in the study.

### Intervention Development Approach

#### Overview

**Figure 1:**
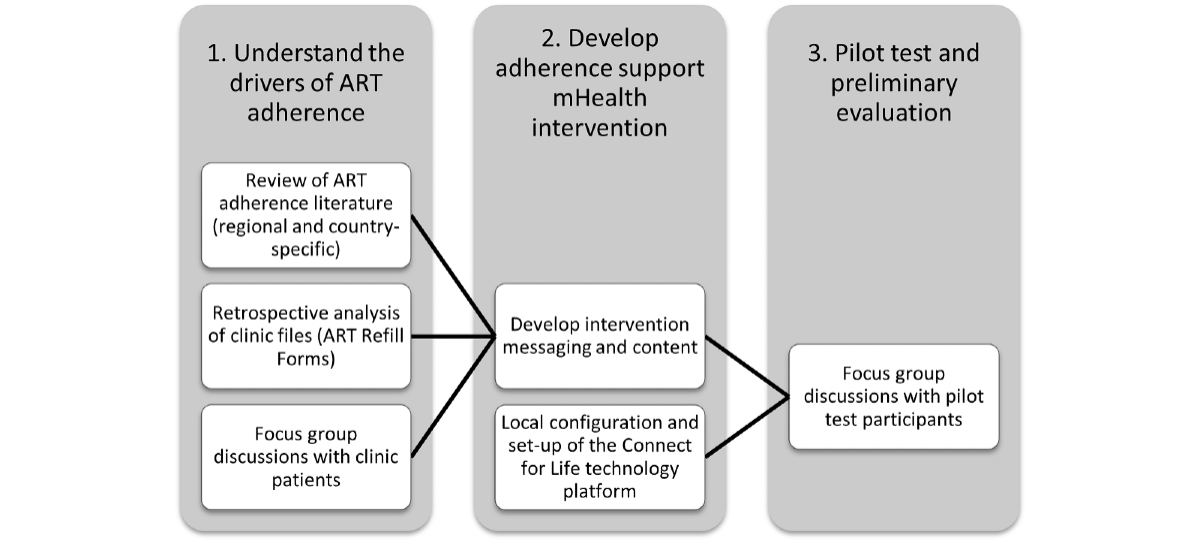
Intervention development process. ART: antiretroviral therapy, mHealth: mobile health.

To determine the best configuration of mobile phone support services for patients in the study site, we used the following methodology (Figure 1):

1. Formative research to understand the drivers of ART adherence: literature review of factors associated with ART adherence (global, regional, and country-specific data), retrospective analysis of clinic files (ART refill forms), and focus group discussions (FGDs) with patients in the SHIP clinic.
2. Development of mHealth intervention: we adapted an existing mHealth adherence support platform, tailored it to the setting and target population, and pilot-tested the platform, guided by the Behavior Change Wheel (BCW) approach.
3. Pilot test and preliminary evaluation: we piloted the intervention with a subset of clinic patients for 8 weeks and then conducted FGDs with patients who received the intervention in the pilot phase.

#### Formative Research to Understand the Drivers of ART Adherence

##### Review of ART Adherence Literature

A literature review of regional, country-specific, and site-level routine clinical data on ART adherence was conducted by the investigators. The literature provided point estimates for adherence that could serve as a comparison with our study population and outline some of the main facilitators of and barriers to adherence in the Philippines context.

##### Retrospective Analysis of Clinic Files

A record review of all pharmacy refill forms from the study clinic was conducted. Data were captured from 3381 pharmacy refill forms collected during routine clinical care for 682 patients between May 2012 and August 2016. The pharmacy refill forms included basic demographic information, dispensing data, pill count, and self-report of the number of doses missed in the past 30 days. Data quality for these forms was poor, with missing forms, fields left blank, and inconsistent or conflicting data in a large proportion of the records. Owing to these limitations in data quality, only the most recent refill form for each patient was included in the analysis, as data were much more complete in the recent forms. The estimate of adherence was calculated as follows:

Adherence percentage = 1 − (number of pills reported missed since last visit/number of pills dispensed at the last visit) **(1)**

##### FGDs With Clinic Patients

During the formative research stage, the study team conducted FGDs to explore adherence challenges and possible approaches to support adherence. The specific topics covered during the discussions were adherence challenges, use of mobile phones, attitudes toward receiving adherence reminders, priority health education topics for mHealth tips, and acceptability of receiving an adherence score as a feedback mechanism.

Focus group participants were recruited through convenience sampling of clinic patients as identified by the SHIP clinic physician. Patients were eligible to participate if they were aged ≥18 years, HIV-positive and on ART at the SHIP clinic, and willing to participate in a group discussion setting. Privacy around HIV-positive status was the biggest barrier to recruitment, and only patients who were publicly open about their HIV-positive status participated in FGDs.

Each FGD was facilitated by a qualified HIV test counselor who was experienced in qualitative methods. A second staff member took detailed notes throughout the session, and immediately after the discussion, the notetaker and facilitator debriefed and recorded their initial observations. The FGDs were conducted in a mix of English and Filipino, which is common in Metro Manila. The discussions were held in a hired conference room located in the building next to the SHIP clinic, selected for the convenience of the participants. Before the discussion, the participants completed the informed consent process and provided demographic data and ART adherence data using a short questionnaire. The FGDs were audio recorded on 2 devices, and the discussions ranged from 60 to 105 minutes.

The FGDs were transcribed, and a framework-guided rapid analysis was conducted. Transcripts were manually coded using a deductive coding methodology in which initial coding grouped responses into overarching themes as per the topic areas included in the FGD guide. Following initial coding, line-by-line coding was used to assign the subthemes. Qualitative data were consolidated in a structured template based on the a priori research questions. The template enabled the consolidation of data into matrices by each category to identify salient themes.

#### Develop Adherence Support mHealth Intervention

##### Develop Intervention SMS Text Messaging and Content

The intervention development process was broadly guided by the BCW developed by Michie et al [43-45]. Behavior change techniques (BCTs) related specifically to ART adherence were informed by the information-motivation-behavioral skills (IMB) model of ART adherence [46].

**Figure 2:**
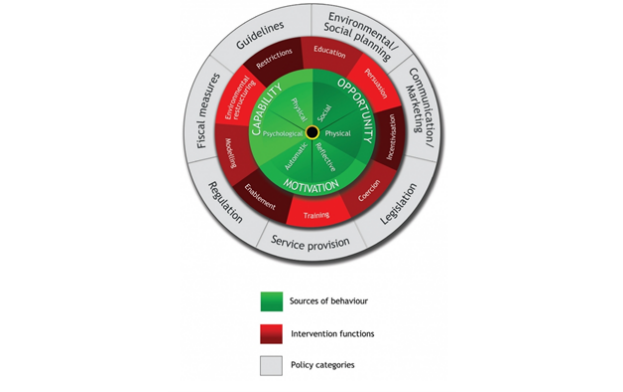
The Behavior Change Wheel [45].

The BCW is a method for characterizing and developing behavior change interventions based on a comprehensive causal analysis of behavior (Figure 2) [45]. In the BCW approach, the intervention design process consists of 3 stages.

The first stage of intervention development is to understand the behavior. In this case, the specific target behavior is optimal adherence to ART, defined as taking at least 95% of the prescribed ART doses on time. To understand behavior, the BCW approach starts with the question, *What conditions internal to individuals and in their social and physical environment need to be in place for a specific behavioral target to be achieved?* On the basis of the formative research findings, the components of capability, opportunity, and motivation that interact to account for behavior in the BCW approach were summarized [43-45]. Using the capability, opportunity, motivation, behavior (COM-B) model, we aimed to understand the challenges faced by patients and identify opportunities to address specific behaviors through the provision of BCTs.

The second stage of intervention development in the BCW model is to identify the intervention options. In this case, we planned to use an mHealth platform that would be tailored to the setting and population.

**Figure 3:**
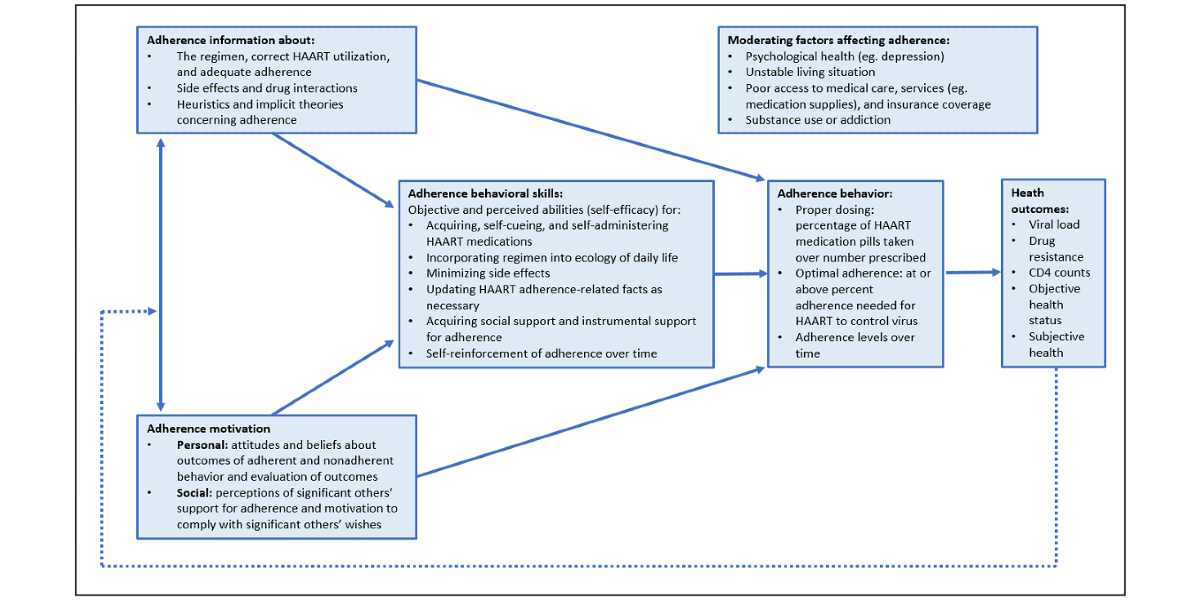
The information-motivation-behavioral skills model of highly active antiretroviral therapy adherence [46]. HAART: highly active antiretroviral therapy.

The third stage is to identify the content and implementation options, including BCTs and mode of delivery. To better understand the most appropriate BCTs, we referenced the BCW taxonomy of BCTs [47] and IMB skills model of ART adherence (Figure 3) [46]. IMB is a useful behavioral theory for exploring factors that lead to adherence and is supported by robust evidence [46,48,49]. It posits that adherence-related information, motivation, and behavioral skills are the fundamental determinants of adherence to ART. The model’s mediational assumption asserts that ART adherence information and motivation generally work through ART adherence behavioral skills to affect adherence behavior. We used the IMB skills model to identify the aspects of motivation, information, and behavioral skills that our intervention might target.

**Figure 4:**
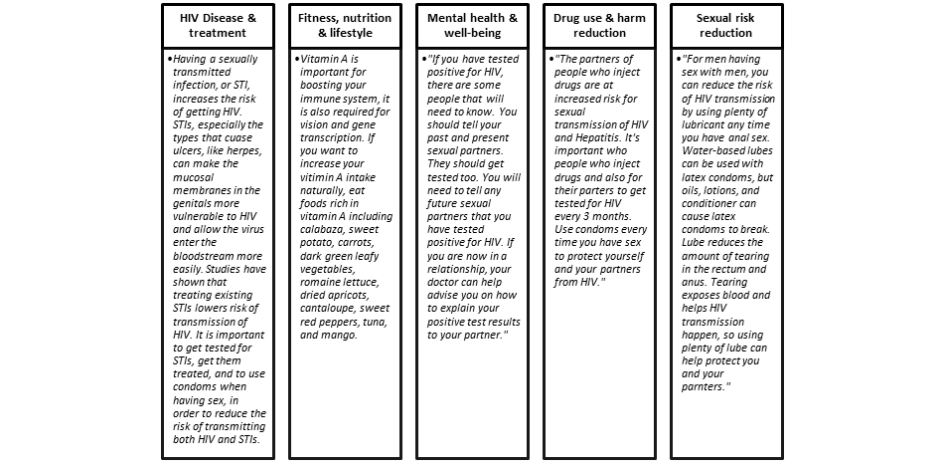
Health tip topic areas and sample tips. STI: sexually transmitted infection.

The intervention services were tailored based on input from the IMB skills model, input by SHIP patients during FGDs, and information from clinical service providers at the study site. The study team and clinicians worked together to write 210 health tips, script the reminder messages, and map the call flows. The lead clinic physician created a symptom-reporting algorithm. A local voice talent agency was engaged to record the content. Figure 4 provides examples of tips in each of the health tip categories.

##### Local Configuration and Setup of Connect for Life Technology Platform

From 2015 to 2016, SHIP staff worked with internet technology specialists and public health professionals from the study sponsor Janssen Global Public Health to adapt the Connect for Life platform for use at the SHIP clinic. Connect for Life is a technology built on the Mobile Technology for Community Health (MOTECH) open-source software platform [50]. It enables health facilities to connect to patients via their cell phones or feature phones through IVRS or SMS text messaging. It was piloted in India and Uganda before roll out in the Philippines [51,52].

The platform has the following functionalities: pill reminders, visit reminders, symptom reporting, health tips, and adherence feedback messages. The study team collaborated with clinic physicians and software developers to adapt the various functions of the Connect for Life platform to align with the needs of the patients, as documented in the formative research phase.

#### Pilot Test and Preliminary Evaluation

##### Overview

During the first 8 weeks of piloting the intervention, 62 patients were enrolled in the study. These patients received adherence reminder calls and health tips and reported their adherence via IVRS. During this pilot test phase, the feasibility and acceptability of the intervention were analyzed before moving to a larger scale implementation phase.

##### Feasibility

To assess the feasibility of the intervention, use data from the Connect for Life platform were analyzed. This included the number of calls generated from the platform, the number of calls answered by the participants, and the outcomes of those calls.

##### Acceptability

To assess acceptability, 2 FGDs were held to assess user experience. All eligible study participants were invited to participate in the focus groups, of which only 5 agreed to participate in a FGD (the major barrier to participation in these FGDs was the difficulty of transport owing to the traffic congestions in Metro Manila). Participants discussed their experience with Connect for Life; their reactions to the reminders, health tips, and adherence feedback; their feedback on the call length and call frequency; and their suggestions for improving the system.

## Results

### Understanding Drivers of ART Adherence

#### Review of ART Adherence Literature (Regional and Country-Specific)

Globally, approximately 40% of patients report suboptimal adherence to ART [53,54]. In the regional Therapeutics, Research, Education, and AIDS Training (TREAT) in Asia cohort (which includes 12 clinical sites from Thailand, Hong Kong, Malaysia, the Philippines, and Indonesia) of the 1316 patients, 421 (31.99%) self-reported suboptimal adherence of <100% [13]. Similar to our Connect for Life study cohort, majority of the TREAT Asia group comprised a male (67%) population and was aged <40 years (66%); however, most participants of the TREAT Asia cohort were exposed to HIV via heterosexual contact (69%), whereas our study group was primarily homosexual. The study found that the adherence rate was the lowest during the first 6 months on ART and the rate improved the longer the patient was on treatment [13].

Several key factors influencing ART adherence are well documented in the literature, including medication side effects, substance abuse, presence of social support, and time spent on treatment [13,28,54-57]. In the Philippines context, issues of stigma and discrimination also emerged as a major barrier to medication adherence [1,58,59].

#### Retrospective Analysis of Clinic Files

On the basis of the pharmacy refill forms for SHIP clinic patients, 67.7% (317/468) of patients reported perfect adherence in the 30 days before their most recent refill, 31.8% (149/468) reported suboptimal adherence <100%, and 20% (94/468) reported adherence <95%. A retrospective review of pharmacy refill data is summarized in Table 1.

**Table 1:** Sustained Health Initiatives of the Philippines clinic adherence data from pharmacy refill forms (N=682).

				Value
**Demographic information (n=542),** **median (IQR; range)**				
	Age (years)		32 (28.6-35.9; 21-72)	
	**HIV history (years)**			
		Time since diagnosis	3 (1.8-5; 0-25)	
		Time from diagnosis to antiretroviral therapy initiation	0.2 (0.1-0.9; 0-21)	
		Time on antiretroviral therapy	2.4 (1.5-3.9; 0-10)	
**Adherence estimates for patients with 30-day adherence reported at the last pharmacy refill (n=468), n (%)**				
	100% adherence		317 (67.7)	
	Missed 1 dose—adherence (95%-100%)		55 (11.8)	
	Missed ≥2 doses—suboptimal adherence (<95%)		94 (20.1)	

#### FGDs With Clinic Patients

We also conducted FGDs with 1.8% (12/682) of the participants regarding their adherence challenges. All participants were male, 75% (9/12) were homosexual, 25% (3/12) were bisexual, and 67% (8/12) had full-time employment. The time patients had been on ART ranged from 5 months to 6 years, with a median time of 4 years. Overall, 83% (10/12) of the participants reported that they sometimes forgot to take their medications and 42% (5/12) had missed a dose within the past 2 weeks.

FGD findings on the causal factors for ART adherence are summarized in Table 2.

**Table 2:** Causal analysis of antiretroviral therapy adherence behavior.

Reason for nonadherence		Illustrative quotes from FGD^a^ participants
**Inconsistent daily routines, change in habits, behavioral skills, or difficulties with timing of dose**		
	Common reasons that patients report missing doses include simply forgetting, being busy, being away from home, and changes in routine [57].	“You usually...take it at home, not in the office; there are some instances when you calculated the time...so you have to be in the office to take it properly. Then when you are there, you forget to take it, it’s because you’re busy already working.” “The challenge that I faced with ARV^b^...I think it’s very essential for those working in BPO [business process outsourcing], is adjusting the time when your schedule shifts, because it has to be taken during your sleeping time...And, you know, you can’t disclose, ‘I was late because I overslept because I was really high with my ARVs’.”
**Low social support**		
	Patients who have a treatment support person are more likely to be adherent [60]. Having a good relationship with the HIV primary care physician and clinic staff was an important factor.	“My partner is really helping me a lot to adhere to the schedule in taking the medications...When my partner gets too busy, the tendency is that we both forget that I need to take the medications.” “The reason why most of the patients are lost to follow up is because they feel like they are treated like patients in other [HIV treatment] hubs. The reason why we continue going to SHIP is because we feel welcome, we feel like it’s like an extension of our family. Unlike in other hubs – they feel they have to wait; they don’t know if they are going to die on that day or that hour. They feel that they are not that important.”
**Medication side effects or type of regimen**		
	Experiencing an adverse drug reaction is associated with poor adherence [61]. Furthermore, a large cohort study in Southeast Asia found that patients taking an NRTI^c^ + NNRTI^d^ regimen had poorer adherence than those who initiated on an NRTI + protease inhibitor regimen [13]. This is most likely because of difficulty tolerating the central nervous system side effects of efavirenz, a theme that was noticed throughout our focus groups.	“If we open a fresh bottle of ARV sometimes it feels kind of strong...It’s like the first time. You feel all the side effects of the ARV.” “For me it really is the headache, especially this first few weeks.” “Especially when I was having a pneumonia, especially with interactions with antibacterials – It’s really hard to actually take the ARV together with the other medicines because you will be getting a really, really painful stomach, even if you ate something. So sometimes in order for me to finish the whole course of the meds that’s been described I have to skip if I really can’t tolerate anymore.”
**Shorter time on ART**		
	Some studies show that longer duration on ART^e^ is associated with better adherence [13]. Treatment-experienced FGD participants insisted that *newbies* would benefit most from the intervention.	“For the newbies this would be a big help because for a while it’s a way for them to adjust. Not all of them are still open in discussing their status with people, and this is a first step for them to accept the fact that they have this situation that they need to cope with. And to do that, it’s like the IVR is helping [them]. So, it’s a big help.”
**Substance use or abuse**		
	Patients who use illicit drugs or abuse alcohol may be less likely to adhere to their medication regimen [54,62]. Among our focus group participants, use of methamphetamine in the context of *Partee n Play* emerged as a theme.	“[When you are high on drugs] You tend to delay it more and more. When you are high you are more carefree, it’s like ‘I’ll take it later, then later, then later’…” “I make it a point of, I have been with my friends taking drugs, and then I know that some of them have that schedule of taking the ARV. So I make it a point that I remind them to take ARV. It’s like a sisterly bond, like ‘Friend, it’s your time…’ You have to insist. It’s like a responsibility within friends.”
**Stress, coping abilities, or poor mental health**		
	People living with HIV are more likely to be affected by depression and anxiety [54,55]. Focus group participants stated that coping with a new diagnosis can be overwhelming. Interruptions in treatment for patients who have been in care for several years may be caused by episodes of depression.	“The only reason why we really skip for days is like when you are really depressed. And drugs, with your serotonin and dopamine levels really low and you’re really emotional. You tend to be like ‘my life sucks and I don’t want to take my meds.’” “You mentioned harm reduction – okay, yes. Could be. Another thing we are not really addressing is mental wellness...It’s one reason why we consciously skip our medication, is our mental wellness.”
**Stigma**		
	Many people living with HIV are fearful of the repercussions of disclosing their status (or having it disclosed inadvertently) to family, friends, or employers [33,55,63,64]. Focus group participants shared their fears and their experiences that disclosing their diagnosis could result in personal rejection, losing their housing (multi-generation family homes are the norm in Philippines), or being dismissed from their jobs. They do not want to be seen taking medicines around other people. The psychological challenges of coping with and accepting an HIV diagnosis during the early stages is a major factor for nonadherence.	“For me I’ve been battling this on my own for 6 years. None of my relatives know that I’m positive. The only people that know that I’m positive are my friends. So, I think this reminder thing...the IVR thing, the health tips, is really good.” “I think there is one point that I when I consciously, not really skipped, but delayed it 4 to 6 hours, just because when that alarm went really crazy everyone was looking at me...There’s this thing now that gay people are being judged when we take our meds in public...That’s why it’s hard to have that really loud alarm now.”

^a^FGD: focus group discussion.

^b^ARV: antiretroviral.

^c^NRTI: nucleoside reverse transcriptase inhibitor.

^d^NNRTI: nonnucleoside reverse transcriptase inhibitor.

^e^ART: antiretroviral therapy.

### Development of the mHealth Intervention

#### Overview

The intervention services were tailored based on the input from SHIP patients and clinicians during formative research.

**Figure 5:**
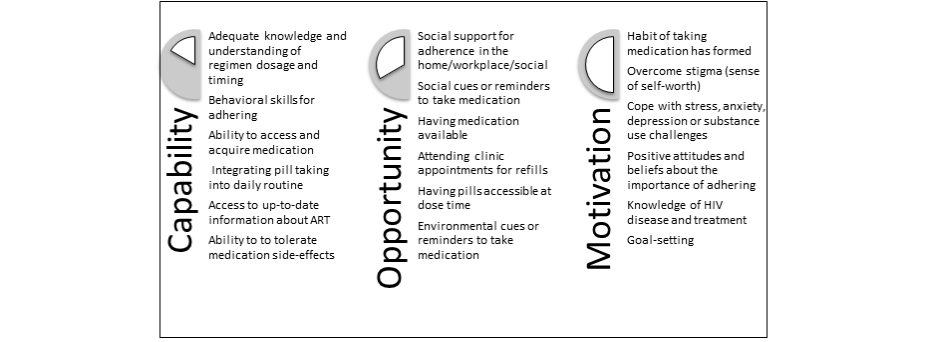
Summary of the components contributing to optimal antiretroviral therapy adherence based on the capability, opportunity, motivation, and behavior and information, motivation, and behavioral models. ART: antiretroviral therapy.

On the basis of the formative research findings, the various components of the COM-B framework were summarized, incorporating the aspects of the IMB skills model of adherence (Figure 5).

The focus group findings suggested that mobile phones would be an acceptable mode of delivery for HIV interventions targeting young MSM in the Philippines. During the FGDs, participants provided detailed input about the acceptability of various intervention aspects, including pill reminders, health tips, visit reminders, adherence feedback, and symptom reporting.

#### mHealth Intervention Preferences

Patients reported that they would like mHealth services to be personalizable. For example, patients requested to be able to select whether they receive pills and visit reminders via SMS text messages or via calls, as well as to determine the frequency and the time of the day that they receive reminders. Participants believed that newer patients who recently started ART would benefit most from daily pill reminders and experienced patients would prefer less frequent reminders. They stated that it would be important to be able to opt in or out of any call or SMS text messaging services at any time.

Patients expressed a strong interest in health tips that covered a variety of topics, not strictly HIV disease, and they wanted to personally select which categories of health tips they would hear.

In summary, patients suggested that an ideal mHealth service should be personalized based on the following factors: (1) call or SMS text messages, (2) timing of calls or messages, (3) frequency of calls or messages, and (4) content or topic areas for health education messages.

#### mHealth Intervention Configuration

The investigators created a standard service scheme (Table 3) that could be adjusted at the patient’s request. The recommended service scheme included *pill reminders* for all patients. As focus groups and literature review suggested that more intensive adherence support is required in the early stages of HIV treatment while forming of habits, ART-naïve patients and patients in their first 24 weeks of ART received reminders daily for the first 24 weeks and weekly for the next 24 weeks. Patients who were experienced with ART received weekly reminders for 24 weeks and no reminders after 48 weeks.

During IVRS pill reminder calls, patients were prompted to *report symptoms or side effects* of medications using an IVRS touch-tone menu. The patients received SMS text message recommendations for over-the-counter medications and advice depending on the algorithm outcome. The system automatically generated an alert for the clinician of any symptom reports that required urgent attention.

All patients received SMS *appointment reminders* at 2 set times in advance of their scheduled clinic visit date.

All patients who received IVRS calls for their pill reminders could receive a weekly *adherence feedback* message informing them of their *score*—from 0 to 7—based on the number of days they reported taking their doses in the prior week via the IVRS platform. The adherence feedback score was followed by a short motivational message to encourage improvement among patients with low adherence or support continued good adherence among patients with high adherence scores.

Patients would automatically receive audio *health tips* when they received pill reminder calls, or they could opt to receive health tips via SMS text messages. For patients new on ART, there was a tailored set of health tips that explained the basics of HIV and ART. In addition, we created tips on a variety of other health topics based on the suggestions of patients from the FGDs. The following five broad categories were selected for health tips: (1) HIV disease and treatment, which include tips about HIV testing and diagnosis, transmission of HIV, coinfections, and laboratory tests for people living with HIV; (2) fitness, nutrition, or lifestyle, which included tips for exercise and eating healthy; (3) mental health or well-being, which included tips on acceptance and disclosure of HIV status, and approaches for understanding and dealing with depression, anxiety, and stress; (4) drug use and harm reduction, which included medical information about common recreational drugs, safer injection, and hepatitis C; and (5) sexual risk reduction, which included tips on condoms and lubricants and tips on leading a healthy sex life with HIV.

The investigators worked with 2 clinic providers and a local voice talent agency to write and record 210 health tips that related to the common questions and issues raised by patients and tips that incorporated the themes that emerged from the focus groups. The messages were crafted ensuring that they not only provided didactic information related to the health topic but also ended with a specific action or behavior that the patient could adopt to improve or to minimize the impact of a specific behavior.

The system was configured to protect patient privacy and prevent unintended disclosure of health information. Upon answering any call from the system, the patient would immediately hear a *jingle*, a song that was associated with the Connect for Life system. Upon hearing the *jingle*, they would enter a personal identification number to advance to the next step of the call. No health-related information would be transmitted unless the personal identification number was keyed in, to protect patient privacy and confidentiality.

Services in the intervention package address the 3 main components of the COM-B model. Capability is addressed through health tips, which aim to improve knowledge regarding ART and HIV disease and improve behavioral skills. Opportunity is addressed through the pill reminder service, which provides an external prompt or cue for pill taking and supports habit forming through the appointment reminder service, which prompts attendance at the clinic for refill, thereby increasing accessibility and availability of medications; and through the symptom-reporting algorithm, which addresses the medical barriers to pill taking by expediting a response to side effects or medication reactions. Motivation is addressed through health tips (eg, messages designed to help with stress, overcome stigma, and inform positive attitude toward pill taking) and adherence feedback messages, which reward and reinforce high adherence and encourage improvement for low adherence.

Table 3 presents the proposed service scheme. However, the services were flexible and a patient could opt out of any call or SMS text messaging service that they did not wish to receive or opt into services depending on their preference and the clinician’s judgment. The clinician could reactivate or extend the pill reminders for patients who needed additional support.

**Table 3:** Connect for Life services scheme.

Patient characteristics	Pill reminder and adherence feedback messages (voice or SMS text messages)	Health tips (voice or SMS text messages)	Appointment reminders (voice or SMS text messages)	Symptom reporting (voice calls only)
Treatment naïve and recently initiated (<6 months on antiretroviral therapy), or treatment experienced more than 6 months with adherence <80% in the 30 days before enrollment	Daily reminders from 0 to 24 weeks Weekly reminders from 25 to 48 weeks	Health tips play during all pill reminder calls; health tips topics tailored to new patients	Yes	Yes (during all pill reminder calls)
Treatment experienced >6 months with adherence ≥80% in the 30 days before enrollment	Weekly reminders from 0 to 24 weeks No reminders from 24 to 48 weeks	Health tips frequency and topics selected based on the preference of clinician and patient	Yes	Yes (during all pill reminder calls)

### Pilot Test and Preliminary Evaluation Findings

A pilot test phase was conducted from October 2017 to January 2018, in which 62 patients were enrolled in the service. During the pilot test period, we received reports of several technical issues that affected the functionality of the system. In all, 2 FGDs were held in January and February 2018, after approximately 3 months of the pilot project implementation. There were 5 participants—3 in one discussion and 2 in the next. FGD findings on the themes that emerged from the pilot test are summarized in Table 4.

On the basis of the findings from the pilot phase FGDs, enrollment in the study was suspended because of pending solutions to technical issues. The study team worked with software developers to trace the source of the technical issues. It was determined that the platform was functioning well and that the technical failures were because of issues within the local telecommunications infrastructure (ie, poor call quality). After the team addressed all the technical issues on the software development side, enrollment continued with SMS text messaging services only.

Since mid-2019, we have found that as telecom services improved in the Philippine setting, voice calls in the Connect for Life system can now be delivered with fewer technical issues. Following the initial pilot study, the intervention was scaled up at the SHIP clinic and currently serving 1491 patients at 2 HIV clinics. The platform is being further developed to move from the MOTECH base to open medical record system. We plan to pilot test the new version in several HIV treatment sites across the Philippines.

**Table 4:** Themes emerging from the pilot test evaluation.

Themes		Illustrative quotes from FGD^a^ participants
**CfL^b^ technical issues or functionality**		
	PIN^c^ issues: DTMF^d^ is the signal to the phone company that is generated when a user presses a telephone’s touch keys. All FGD participants reported instances in which they attempted to enter their PIN code and the code was not recognized. The frequency of DTMF problems varied widely among the participants. Call origin: Calls are generated from an interactive voice response platform using a United States–based telecom provider. The interactive voice response service provider sets the incoming call number to be displayed as the patient’s own phone number. However, patients reported that this was inconsistent and that some of the incoming calls from the CfL system that they answered displayed phone numbers originating in different countries.	“Actually, I just experienced that issue last night [DTMF malfunction]. Sometimes I have been able to enter [my PIN code] and sometimes I haven't. The jingle kept going on, so I kept entering the PIN again and nothing happened, so I just hung up.” “In my case, I think I received thrice already from various locations an unknown number that’s why I didn’t bother answering. One from South Korea, one from US and one from China. The problem is if the number is unknown basically I don’t answer it. I’m just guessing that the number came from CfL.”
**SMS vs voice call preferences**		
	There was mixed feedback about whether SMS text messages or voice calls were more effective or acceptable. Some participants said that the frequency and length of voice calls were too much. Several FGD participants requested to be changed from voice calls to SMS text messages, as texting is more convenient and less intrusive. Others preferred to stay on voice calls as they are more difficult to ignore.	“I think SMS would be nice to have as an option. If at the time the program calls you but you didn’t answer an SMS reminder would be good just to keep in touch.” “I hated the call because I’ve been receiving the calls especially when I’m on my way home in an Uber. If I mistakenly answer it without the headset, the voice will be loud and basically everyone in the Uber would know.” “If you are going to put the schedule of the consultation, I’d rather those to be in text because there’s too much information that I need to remember.” “I think it’s also cultural when people don’t like answering calls. Mostly Asians I know don’t like answering calls. I’m not good at answering calls and most of the people I know don’t also like answering calls especially if the number is unknown and overseas and then you hear this very gloomy guy voice.”
**Adherence feedback *gamification***		
	Participants did not like receiving adherence feedback scores because it was inaccurate and made them feel stressed.	“[The adherence feedback score] has no effect. It has no significance to me.” “For me I don't even care about it because it just stresses me out.”
**Pill reminders**		
	Daily pill reminder calls were not as used as expected by the study team based on the findings from the first 2 focus groups. After the pilot phase, patients reported that, although they like the idea of regular reminder calls, in practice, they are often too busy to answer the calls and report their adherence. The issue of poor uptake of pill reminder calls was further compounded by the technical issues with the entering their PIN code (DTMF issue). Some participants said that the pill reminders did not make a big difference for them as they already had other systems in place to remind them to take their medications.	“It helped. Sometimes I would forget but it would help to remind me because I usually take my pill after work, and after work I’m just so tired, I don’t check the time and sometimes I almost forget because I’m so sleepy.” “I hate to be reminded that I have this condition every single day. I know I need to take it but I don’t need to be reminded every single day that I have to.” “If you call seven times a week that’s a bit irritating for the patient. What the patient can do is have the option to get reminded through text.”
**Health tips**		
	The content of the health tips was useful and informative. All participants wanted to continue to receive health tips. Some participants would prefer SMS text messages rather than voice recordings for the health tips. Some thought the voice recording spoke too slowly; therefore, they would prefer to read it by SMS text messages. One technical issue reported was that sometimes the same health tips were received for multiple days instead of receiving a new tip each day, as intended.	“The health tips are super helpful. Those are the tips about alcohol, and that say you can have sex, you are not prevented but protected. There are even those great tips on eating and what you should eat.” “Just the voice. The girl answering the questions in the health tips is okay. The guy is very depressing.”
**Other findings**		
	Participants were enthusiastic about receiving the automated reminders for their clinic appointments. Participants stated they would have liked a more in-depth orientation or onboarding process at the outset of the intervention. They emphasized the importance of onboarding, setting expectations, and a thorough explanation of the intervention. Not all participants understood they could change or adapt the service model. Peer support: Participants mentioned that they found participating in a FGD with other people living with HIV very helpful and asked if there could be an opportunity for the clinic to organize in person support groups.	“But what I noticed was that it helped with the appointment. That was a big help as I was reminded that I had to go to the clinic. That's a big deal to me. But about missing the meds, it's still human.” “I think the program’s good. I could recommend that for the newbies. I think the program should be laid on properly. For example, scheduling, the time, reminders, and the tips. Maybe after a month if the patient has already established a routine so maybe it could lessen the reminders.” “Besides, the importance of the support group is for patients who have not disclosed to family members. There you can get support or have conversations like this. If there were a support group now, I’d want to be a part of it because I would like to share what I have experienced before with others.”

^a^FGD: focus group discussion.

^b^CfL: Connect for Life.

^c^PIN: personal identification number.

^d^DTMF: dial tone multifrequency.

## Discussion

### Principal Findings

The intervention development approach resulted in an mHealth intervention tailored to the information needs and communication preferences of MSM in the Philippines. The intervention was designed to address various aspects of capability, opportunity, and motivation to achieve optimal adherence to ART.

The formative research found that mobile phone use is widespread in the Philippines and that mobile phones are an acceptable mode of communication for health information and adherence support. The literature review and FGDs revealed that in our patient population, key behavioral barriers to adherence included challenges around forming consistent routines and habits, low social support, stress and mental health issues, substance use, and social stigma of living with HIV. Focus group participants strongly emphasized the need for social and family support to enable and encourage good adherence to ART. Key clinical issues affecting adherence included medication side effects (especially among efavirenz-based regimens) and shorter duration on ART.

Following the pilot test, recipients of the intervention reported that the tone, frequency, and content of the voice messages were acceptable and appropriate. In the prepilot focus groups, participants preferred the male voice actor whose voice sounded more “attractive” according to several participants, whereas in the postpilot groups, several participants mentioned that they preferred the female voice actor because her tone was warmer and she came across as a trusted friend. This finding indicates that more iterations of recording should be tested in future implementations before a full-scale roll out and that budgets and project work plans should allow for several rounds of recording. The accounts of the focus group participants indicated that the intervention increased their knowledge and adherence behaviors. However, a large-scale cohort is needed to assess the intervention’s effectiveness.

In the prepilot focus groups, participants were enthusiastic about receiving voice reminders via phone calls, following their experience of participating in the pilot most participants expressed a preference for SMS text messaging over voice calls. This preference may have been related to the inconvenience of answering phone calls, and it may also have been related to the technical problems experienced with the IVRS. These technical challenges posed a significant challenge to the feasibility of the intervention, and delivery would need to be adapted to allow for SMS text messaging options to achieve full-scale implementation.

### Strengths and Limitations

The strength of the intervention development process was the participatory approach, which included the beneficiaries or users of the potential intervention, clinical service providers, and developers of the technology platform. The views of the target audience were collected during focus groups, which informed the tone, style, frequency, duration, and content of the intervention.

The BCW is a robust intervention development approach that provides a comprehensive understanding of the sources of a behavior, spectrum of intervention functions, and environment in which the behavior occurs. A strength of this approach is the COM-B model at the hub of the BCW. By identifying the capabilities, opportunities, and motivations behind a behavior, we can clearly identify the most relevant intervention approaches and BCTs. This approach allowed us to develop a solid intervention plan that described the technique, mode, and content to address each identified barrier to or enabler of ART adherence.

A weakness of our approach was the sampling and recruitment strategy for the participants in the focus groups. It was a challenge to identify patients who were willing to participate in a group where everyone had a HIV-positive status because many patients were not publicly *out* as people living with HIV, indicating that individual interviews may be an option in future studies. The patients who agreed to participate may not be representative of the wider patient population, introducing a degree of selection bias to the process. The study only included patients >18 years and the patients were almost exclusively male; thus, the findings do not address the distinct needs and challenges of adolescents and women living with HIV. In addition, there was low attendance among those who confirmed their intention to participate in the focus groups. This is reflective of the larger need to provide differentiated models of care in the Philippines, as transportation to the clinic site is not easy in Metro Manila because of traffic congestion.

Another weakness of our approach was that we had an intervention mode in mind—mobile phone—at the outset of the intervention development process. Although there are several key determinants of adherence that the Connect for Life platform can address (ie, knowledge, habit forming, and environmental cues to take medication), there are other factors that the mHealth approach does not address (ie, physical availability of medication and social support).

Notably, the technical challenges experienced in delivering the intervention during the pilot phase made it difficult to assess the true acceptability and feasibility of the planned intervention. Feedback received after the pilot phase focused largely on the mobile phone functionality issues, which then limited the discussion regarding the content and design of the intervention as it was intended to be delivered. Conducting a small pilot phase with a few participants allowed us to identify the problems with functionality and adapt the intervention before scaling up the intervention to the larger cohort; however, a more iterative process with several pilot stages would have been advantageous if budget and timeline had allowed us to do so.

### Comparison With Prior Work

Research on ART adherence has shown that less time on ART is associated with an increased risk of poor adherence [13,65-67]. With this in mind, the intervention was designed with more frequent (daily) pill reminders for patients during their first 6 months on ART and less frequent (weekly) reminders for patients with longer than 6 months on ART. However, after the intervention design was completed and pilots, an analysis of the Philippine cohort found a different trend within the study population, observing that, even before receiving the intervention, newer patients in the Connect for Life cohort tended to be more adherent compared with patients who had taken ART for longer and showed signs of *treatment fatigue* [68]. This highlights the importance of the ability of clinicians to tailor the reminder frequency and other intervention functions based on individual patient needs.

Before this study, 2 other projects using the same technology that the Connect for Life program was built on were implemented and evaluated in India and Uganda. First, a program called Treatment Advice by Mobile Alerts (TAMA), provided people living with HIV in India with daily or weekly pill reminders, adherence feedback, automated algorithms for managing clinical events for patients being initiated on ART, health tips, appointment reminders, and real-time reporting to the clinics of patient interaction with TAMA. Evaluation of the TAMA pilot found that patients gave the platform a high system usability score and gave generally positive feedback about their experience with using the technology. In TAMA, patients could call a toll-free number to access health tips and a clinical event algorithm. Health tips were used by 76% (42/55) of the patients, and automated clinical advice was accessed by 64% (35/55) of the participants in the pilot study. In the Philippines, these functions were available only through outgoing system-generated calls and SMS text messaging because of the prohibitive cost of toll-free inbound telephone lines in the Philippine setting [51,69].

The second project, the Call for Life Uganda program, also found good uptake, acceptability, and positive response to the system. In Uganda, there was a strong preference for interactive voice response over SMS text messages, which was different from the Philippines where participants preferred SMS text messages [70,71].

### Conclusions

Our research found that a mobile phone–based SMS text messaging intervention and IVRS intervention were acceptable to MSM in Manila, the Philippines, and the FGDs suggested that it helped promote ART adherence and appointment attendance. A randomized controlled trial is required to establish the effects of the intervention on the clinical outcomes of HIV care and treatment.

## Abbreviations

ART: antiretroviral therapy
BCT: behavior change technique
BCW: Behavior Change Wheel
COM-B: capability, opportunity, motivation, behavior
FGD: focus group discussion
IMB: information-motivation-behavioral skills
IVRS: interactive voice response system
mHealth: mobile health
MOTECH: Mobile Technology for Community Health
MSM: men who have sex with men
SHIP: Sustained Health Initiatives of the Philippines
TAMA: Treatment Advice by Mobile Alerts
TREAT: Therapeutics, Research, Education, and AIDS Training
